# Novel sonographic reference charts for early pregnancy based on known gestational age

**DOI:** 10.1016/j.xfre.2024.11.008

**Published:** 2024-12-04

**Authors:** Julia Häusler, Alexander M Quaas, Manuel Fischer, Andreas Schötzau, Fiona Pugin, Christian De Geyter, Ursula Gobrecht-Keller, Beatrice Mosimann, Gwendolin Manegold-Brauer

**Affiliations:** a Center for Reproductive Medicine and Gynecological Endocrinology, Department of Obstetrics and Gynecology, University Hospital Basel, Basel, Switzerland; b Shady Grove Fertility, San Diego, California; c Department of Obstetrics and Gynecology, University Hospital Basel, Basel, Switzerland; d Eudox (Statistical Consulting), Basel, Switzerland; e Department of Biomedicine, University of Basel, Basel, Switzerland; f Division for Obstetrics and Prenatal Medicine, Department of Obstetrics and Gynecology, University Hospital Basel, Basel, Switzerland; g Division for Gynecologic and Prenatal Ultrasound, Department of Obstetrics and Gynecology, University Hospital Basel, Basel, Switzerland

**Keywords:** Crown-rump length, first trimester, gestational sac, growth charts, ultrasound

## Abstract

**Objective:**

To study pregnancy dating, we developed growth charts using data from pregnancies with known gestational age. The most widely used growth charts for ultrasonographic early pregnancy variables were developed decades ago and are based on dating by the last menstrual period. The aims of the study were to create growth charts for early pregnancy (including crown-rump length, gestational sac diameter, yolk sac diameter, and amniotic cavity diameter) for last menstrual period-dated spontaneous pregnancies and pregnancies conceived through assisted reproductive technology (ART) with known gestational age; and to compare crown-rump length measurements with two established traditional charts based on last menstrual period dating.

**Design:**

Retrospective cohort study.

**Subjects:**

Of 1,061 singletons, 2,803 crown-rump length measurements from 716 exactly dated ART gestations with 1,143 crown-rump length measurements from 345 spontaneous pregnancies, which were dated by the last menstrual period in women with regular cycles, were compared using nonlinear quantile regression.

**Exposure:**

A total of 716 ART pregnancies and 345 spontaneous pregnancies underwent ultrasound examinations, with a median of four visits at various points during the first trimester.

**Main Outcome Measures:**

Early pregnancy dating with growth charts based on crown-rump length, gestational sac-, yolk sac- and amniotic cavity diameter.

**Results:**

Novel reference charts for ultrasound dating of gestational age based on crown-rump length from 4 + 6 to 10 + 6 gestational weeks were created. The difference of the 10th and 90th percentile was significantly higher in spontaneous compared with ART pregnancies (*P*<.05). For the same crown-rump length, the gestational age of spontaneous pregnancies was estimated to be up to 4.08 days older than in ART pregnancies at the 90th percentile. Preexisting curves (Robinson/Fleming, Hadlock) aligned closely with the new reference curve mid-range but showed larger deviations at the extremes of assessed gestational ages.

**Conclusion:**

Early pregnancy crown-rump length curves based on last menstrual period may overestimate gestational age. Using 2,803 crown-rump length measurements, we propose the current comprehensive charts for early pregnancy within a population with known gestational age for crown-rump length, gestational sac diameter, yolk sac diameter, and amniotic cavity diameter.

Dating pregnancies plays a central role in antenatal care. Early detection of deviations in growth, assessed by early pregnancy biometric variables including the crown-rump length (CRL), gestational sac diameter (GSD), and yolk sac diameter (YSD), is crucial for predicting adverse pregnancy outcomes (1, 2, 3).

Conventionally, the gestational age (GA) is determined by the first day of the last menstrual period (LMP). This method may result in inaccuracies due to imprecise recall of the LMP, as well as variations in the length of the follicular phase and the time taken for conception to occur. Several studies have indicated that LMP dating frequently overestimates GA when contrasted with ultrasound-based dating. This estimation leads to more incorrectly labeled post-term births, which implies that the dating method influences birth-management such as the induction of labor (4, 5, 6, 7).

Therefore, ultrasound examination is the standard for all first-trimester pregnancies (4). Widely used growth charts for the estimation of GA by CRL measurement have been developed decades ago: the growth chart by Robinson and Fleming from 1975 is based on 334 transabdominal measurements with limited resolution with pregnancy dating using the LMP method (8). Another growth chart still used in clinical practice was created by Hadlock 1992 based on 416 pregnancies (9). Considering the advancements in ultrasound technology, this study sought to assess the validity of the existing growth curves using data from a larger patient sample.

Thus, the aims of our study were to assess the influence of the LMP dating method on CRL growth curves by comparing the growth curve for spontaneous pregnancies dated by LMP with the one for assisted reproductive technology (ART) -conceived pregnancies with precise fertilization and implantation dating; to compare novel growth charts with preexisting reference curves; and to complement reference curves with additional biometric measurements such as GSD, YSD, and amniotic cavity diameter (ACD).

## Materials and methods

This retrospective single-center cohort study includes data from 3,946 CRL measurements in 1,061 singleton pregnancies, collected from January 2010 to December 2020. Growth charts were created using data from a median of four visits at different times during the first trimester. All ultrasound examinations were routinely performed at one university fertility center by several trained gynecologists. At the study institution, close monitoring of first-trimester pregnancies with frequent ultrasound examinations until 10 + 6 gestational weeks was routinely conducted. The accuracy of sonographic measurements during early pregnancy was monitored using certified quality control management systems in place (ISO 9001:2000 and ISO 17025). The data were extracted, anonymized, and transferred to a local database for statistical analysis. The study design and reporting of this observational study were based on strengthening the reporting of observational studies in epidemiology guidelines. Before undertaking the study, ethics approval was obtained by the institutional research ethics board (EKNZ, project number 2022-00723). Ethical standards have been adhered to appropriately. Participants who explicitly refused to participate in any research at our study institution were excluded from the study population. When no information about informed consent was available, the ethics committee approved our request not to obtain individual patient consent from this group retrospectively.

**Figure 1:**
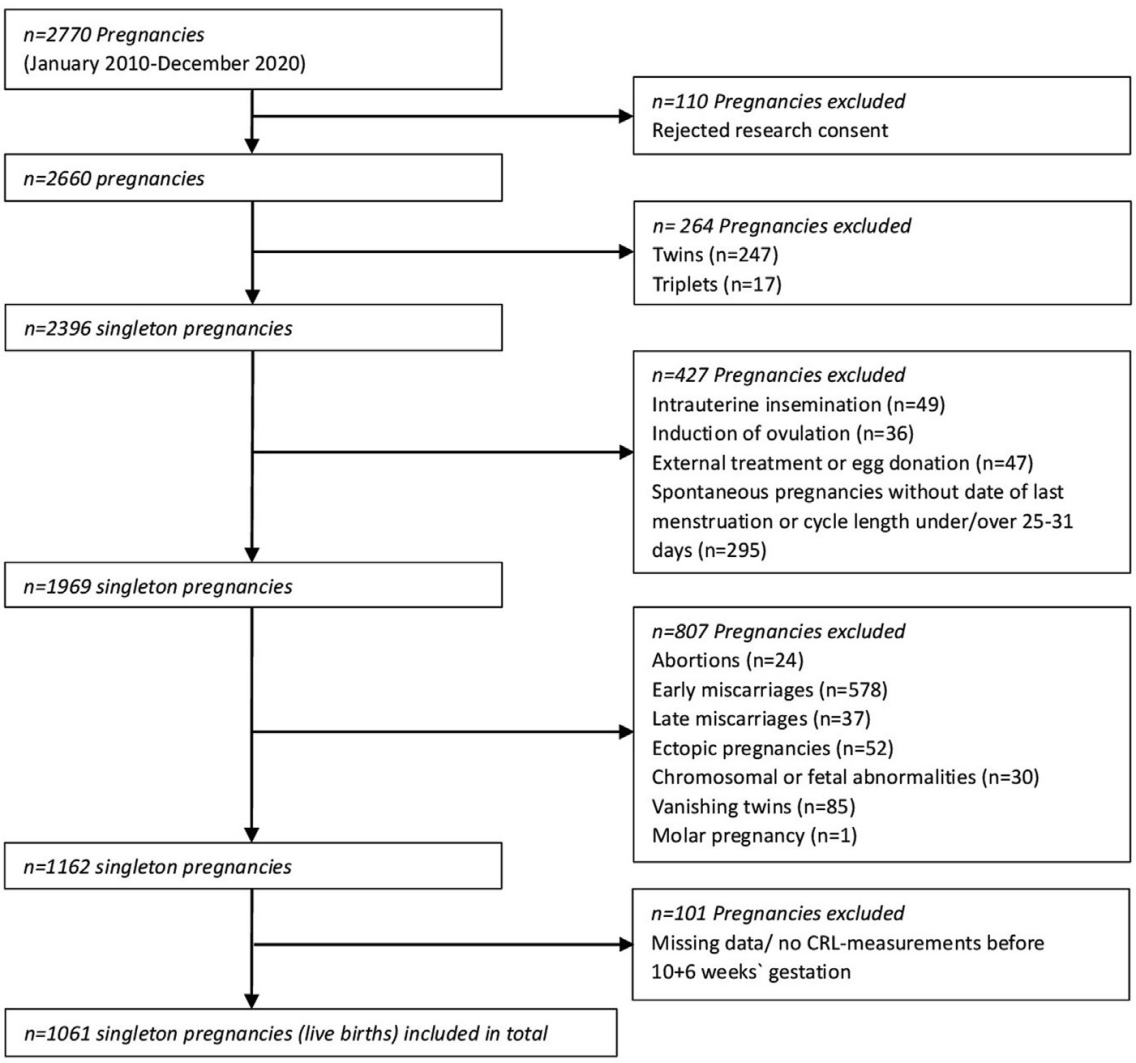
Patient inclusion flowchart. CRL= crown-rump length.

The study primarily included all pregnancies examined between 2010 and 2020, totaling 2,770 pregnancies from which 110 were excluded because of a rejected research consent. In total, 247 twins and 17 triplets were excluded, as well as pregnancies conceived by methods of fertilization like intrauterine insemination (n = 49), induction of ovulation (n = 36), external treatment or egg donation (n = 47) to minimize the heterogeneity of the study population. Because the spontaneous pregnancies should be dated as reliable as possible by the LMP method, we excluded all spontaneous pregnancies without known date of last menstruation or cycle length under 25 or over 31 days (n = 295). Furthermore, we excluded all pregnancies with abortions (n = 24), early miscarriages (n = 578), late miscarriages (n = 37), ectopic pregnancies (n = 52), chromosomal or fetal abnormalities (n = 30), molar pregnancy (n = 1), or the arising phenomenon of vanishing twins (n = 85), meaning those pregnancies who had 2 or 3 amniotic sacs in the beginning. After excluding 101 pregnancies due to missing CRL measurements before 10 + 6 weeks gestation, a total of 1,061 singleton pregnancies were included (Fig. 1). Because the exact determination of GA is crucial for creating reference charts in the first trimester, we ensured an accurate way to determine GA for ART pregnancies: for the fresh cycles including controlled ovarian stimulation, we determined the date of the ovum pick-up as day 14 of gestation and for the frozen cycles, we determined the day of the blastocyst-transfer as day 19.

The examinations were performed using mainly transvaginal transducers (3–12 MHz transducers), and high-resolution machines (GE Voluson E10, E8, E6, S8 [GE Medical Systems, Zipf, Austria]). The accuracy of the measurements was examined annually by comparisons with measurements of standard structure sizes embedded in a gel. Standard ultrasound caliper application methods for early pregnancy sonography were used as reported in existing literature (10, 11).

Statistical analyses were conducted using the statistical software R, version 4.2.1 (Vienna, Austria, 2022). Significance levels were set at *P*<.05. To assess the relationship between GA and the ultrasonographic biometric variables, nonlinear quantile regression was applied. With the quantile regression model the median, 10th, and 90th percentile of the outcome variable distribution were estimated. Quantile regression was performed using a 5-knot restricted cubic spline for each quantile (12). This model was applied for predicting CRL and other biometric variables (GSD, YSD, ACD) based on GA. To build a novel CRL growth chart based on the ART pregnancies, and subsequently compare it with preexisting charts such as Robinson/Fleming (8) and Hadlock (9) based on LMP, CRL as the outcome variable was predicted by GA. To predict GA using CRL, it is not sufficient to simply exchange the abscissa and ordinate (13, 14). Instead, a different regression equation was used with CRL as the predictor variable to estimate GA quantiles. This chart was compared with that of spontaneous pregnancies to assess the impact of the LMP method on GA estimation.

## Results

From January 2010 to December 2020, 3,946 serial CRL measurements from 1,061 singleton pregnancies were taken to develop novel growth charts. The participants underwent a median of four ultrasound examinations between 4 + 6 and 10 + 6 gestational weeks. Among the 1,061 pregnancies, 345 were spontaneous and dated using the LMP method in women with regular menstrual cycles lasting between 25 and 31 days. A total of 716 were ART pregnancies with known timing of the date of fertilization and/or embryonic stage at embryo transfer, consisting of 381 fresh- and 335 frozen-embryo transfer cycles. Table 1 presents the descriptive statistics of the study population. Growth curves were plotted for both spontaneous (n = 345, 1,143 CRL measurements) and ART pregnancies (n = 716, 2,803 CRL measurements).

**Table 1:** Descriptive statistics of study population characteristics, including median [min, max] for continuous variables, and frequency (%) for categorical variables.

Characteristic	ART pregnancies (n = 716)	Spontaneous pregnancies (n = 345)	n	*P* value
Number of ultrasound examinations/pregnancy	4.00 [1.00; 10.0]	4.00 [1.00; 9.00]	1,061	<.001
Maternal age (y)	34.7 [22.1; 44.4]	35.5 [21.1;48.7]	1,061	.009
Body weight (kg)	62.9 [41.2; 132]	63.0 [43.2; 122]	1,019	.560
Body size (cm)	166 [147; 186]	166 [150; 185]	1,015	.158
Maternal BMI (kg/m^2^)	22.8 [16.6; 48.8]	22.6 [16.8; 41.9]	1,016	.834
Pulse (bpm)	72.0 [41.0; 112]	71.0 [46.0; 118]	1,015	.239
Systolic blood pressure (mm Hg)	120 [89.0; 199]	119 [81.0; 165]	1,016	.442
Diastolic blood pressure (mm Hg)	72.0 [45.0; 106]	72.0 [44.0; 95.0]	1,016	.805
Female causes of infertility				
Uterine factor	54 (7.71%)	47 (17.8%)	964	<.001
Tubal factor	79 (11.3%)	13 (4.92%)	964	.004
Ovulatory factor	102 (14.6%)	28 (10.6%)	964	.133
Ovarian insufficiency	112 (16.0%)	60 (22.7%)	964	.019
Endometriosis	51 (7.29%)	15 (5.68%)	964	.462
Recurrent pregnancy loss	12 (1.71%)	36 (13.6%)	964	<.001
Unexplained infertility	380 (54.3%)	99 (37.5%)	964	<.001
Others	6 (0.86%)	2 (0.76%)	964	1.000
Missing information	16 (2.23%)	81 (23.5%)	1,061	<.001
Fertilization type				<.001
ICSI	535 (74.7%)	0 (0.00%)	1,061	
IVF	181 (25.3%)	0 (0.00%)	1,061	
Spontaneous conception	0 (0.00%)	345 (100%)	1,061	
ART, fresh embryo transfer	381 (53.2%)	0 (0.00%)	1,061	
ART, frozen-embryo transfer	335 (46.8%)	0 (0.00%)	1,061	
Newborn data				
Weight (g)	3,290 [600; 4,930]	3,345 [880; 4,520]	1,033	.363
Head circumference (cm)	34.6 [21.0; 39.0]	35.0 [24.3; 38.0]	961	.451
Height (cm)	50.0 [25.0; 58.0]	50.0 [34.0; 57.0]	1,006	.712
Arterial pH	7.28 [6.69; 7.44]	7.29 [6.74; 7.47]	878	.033
Gestational age at delivery	39 + 3 [23 + 2; 42 + 0]	39 + 4 [25 + 3; 42 + 0]	1,021	.339

ART = assisted reproductive technology; BMI = body mass index; ICSI = intracytoplasmic sperm injection; IVF = in vitro fertilization.

**Figure 2:**
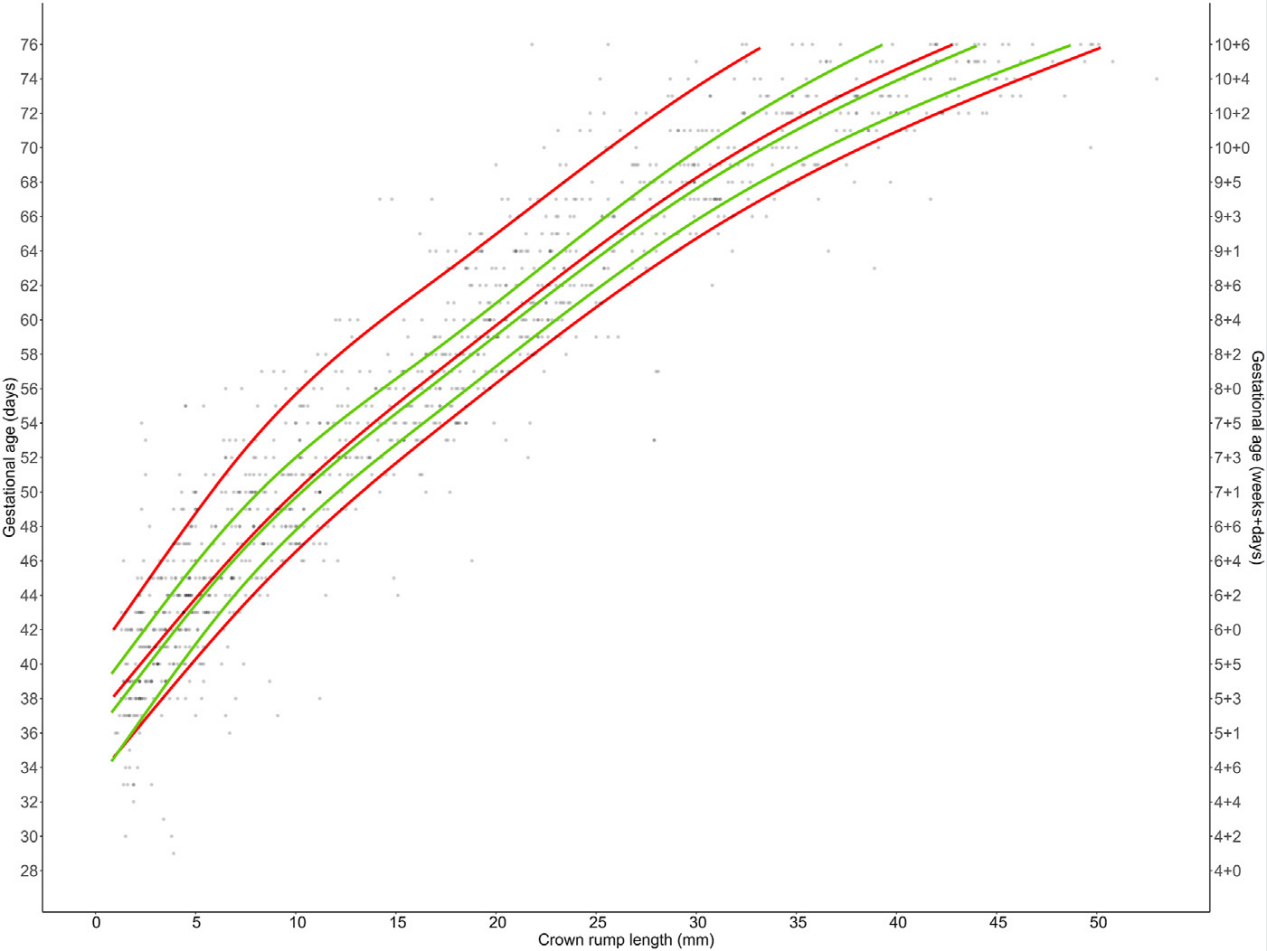
The 10th, 50th, and 90th percentiles of the predicted gestational age based on crown-rump length measurements, for both assisted reproductive technology pregnancies (green) and spontaneous pregnancies (red). Individual data points for spontaneous pregnancies are plotted as gray dots.

The new CRL growth chart for precisely dated ART gestations was compared with the one constructed based on the data collected in spontaneous pregnancies timed by LMP (Fig. 2). The mean difference between ART and spontaneous pregnancies was +1.00 day (*P*<.001) for the 10th percentile and –3.31 days (*P*<.001) for 90th percentile. Thus, the interquartile range for spontaneous pregnancies was larger than for ART pregnancies. The dispersion was more marked toward a higher GA. Differences in GA at the 10th percentile were estimated to be up to 1.25 days lower in spontaneous pregnancies than in ART pregnancies. At the 90th percentile, the GA of spontaneous pregnancies was estimated to be up to 4.08 days higher than in ART pregnancies. The median CRL of spontaneous pregnancies was higher than that of ART pregnancies. The mean difference of CRL between ART and spontaneous pregnancies was –0.55 days (*P*=.014) for the median.

**Figure 3:**
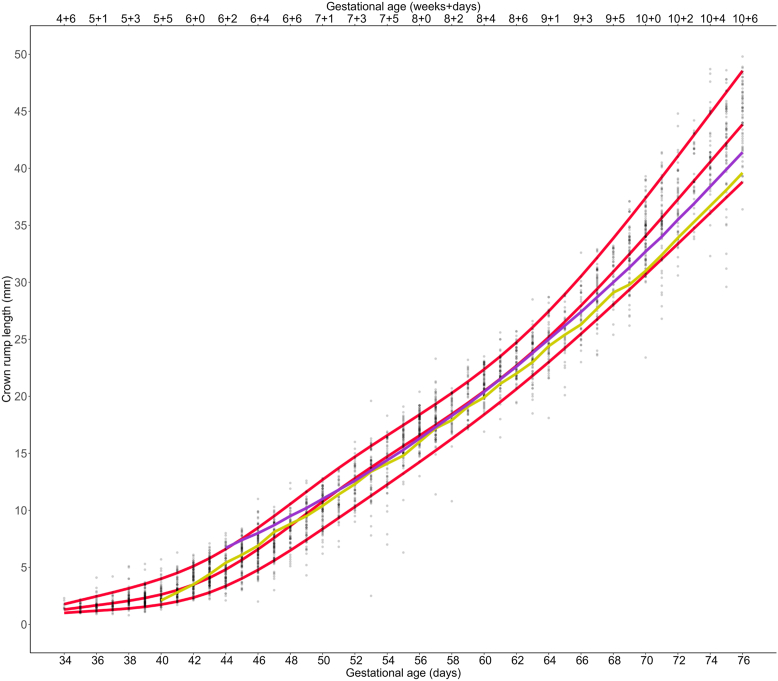
Crown-rump length measurements by gestational age for assisted reproductive technology pregnancies with 10th, 50th, and 90th centiles (red). Existing reference curves inserted for comparison: Hadlock curve (light green) and Robinson/Fleming curve (purple).

**Supplementary Figure 1:**
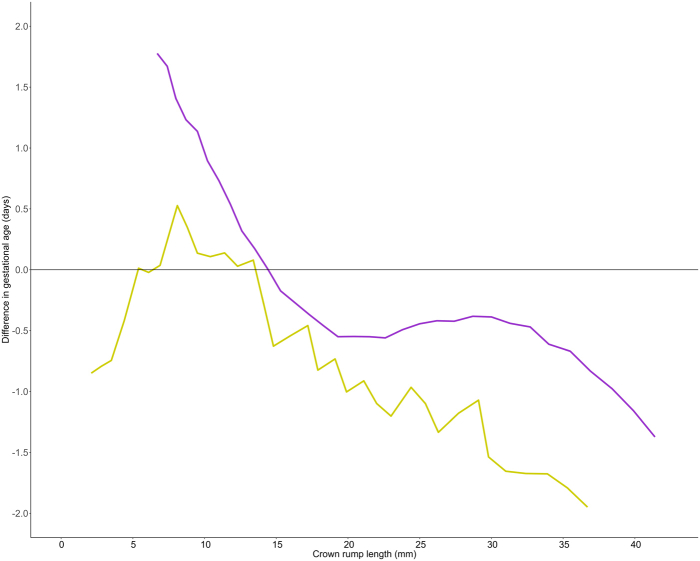
Differences between the predicted median gestational age of the assisted reproductive technology pregnancies compared to the curve of Hadlock (*light green*) and the curve of Robinson/Fleming (*purple*).

The newly developed CRL chart from ART pregnancies was then compared with both the Hadlock and the Robinson/Fleming reference curves (Fig. 3), and a detailed analysis of the variations is presented in Supplemental Figure 1 (available online) (8, 9). Within the mid-range spanning from 7 + 0 to 9 + 0 gestational weeks, the preexisting two curves align closely with the novel ART reference curve. At the beginning of the curve, at a CRL of 2.10 mm, the Hadlock curve deviated 0.85 days from the median and thus, estimated the pregnancy to be more advanced. The curve of Robinson/Fleming started with a CRL of 6.7 mm and estimated the GA 1.78 days lower. At a CRL of almost 40 mm, Hadlock estimated that the GA was 2.32 days higher than the ART reference value at that time, whereas Robinson/Fleming estimated 1.16 days higher. As an example, for a patient with unknown dating presenting with a CRL of 40 mm, our curves would assign a GA of 10 + 4 gestational weeks, whereas Robinson/Fleming would assign more than 10 + 5 gestational weeks and Hadlock would assign more than 10 + 6 gestational weeks.

We created a new CRL reference chart from 4 + 6 to 10 + 6 gestational weeks for ART pregnancies, using known fertilization dates. The chart includes median, 10th, and 90th percentiles for each gestational day (GD) (Supplemental Table 1, available online).

**Supplementary Figure 2:**
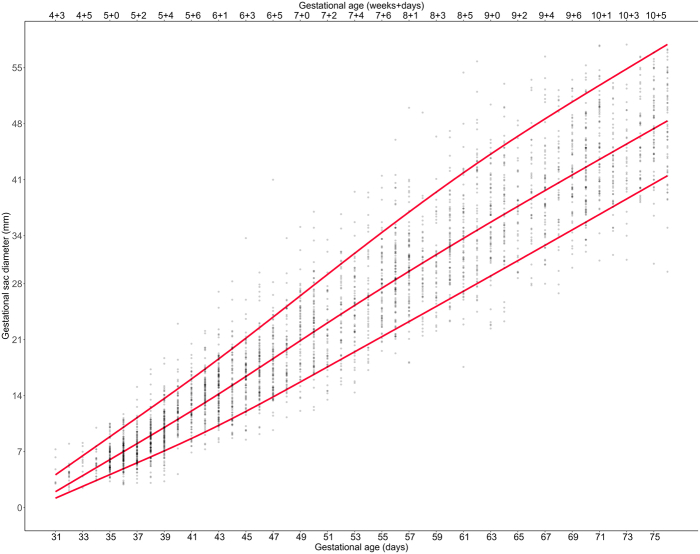
Mean gestational sac diameter measurements by gestational age with 10th, 50th, and 90th centiles for 716 assisted reproductive technology pregnancies (n = 3,190 measurements). *Grey dots* correspond to gestational sac diameter measurements of assisted reproductive technology pregnancies.

This study provided additional growth charts for GSD, YSD, and ACD based on 716 exactly dated ART gestations. Supplemental Figure 2 illustrates scatter plots depicting the 10th, 50th, and 90th percentiles of GSD against GDs. The chart ranges from 4 + 3 to 10 + 6 gestational weeks and is based on 3,190 GSD measurements. Supplemental Table 2 presents reference values (10th, 50th, and 90th percentiles) for GSD based on true GA.

**Supplementary Figure 3:**
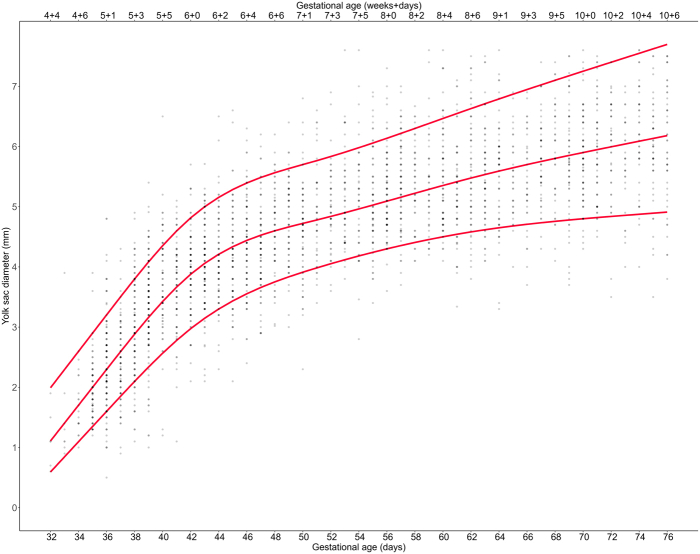
Mean yolk sac diameter measurements by gestational age with 10th, 50th, and 90th centiles for 716 assisted reproductive technology pregnancies (n = 3,001 measurements). *Grey dots* correspond to yolk sac diameter measurements of assisted reproductive technology pregnancies.

Supplemental Figure 3 presents scatter plots that depict the 10th, 50th, and 90th percentiles of YSD in relation to GD. The chart spans from 4 + 4 to 10 + 6 gestational weeks and is constructed from a dataset of 3,001 YSD measurements. Supplemental Table 3 presents reference values (10th, 50th, and 90th percentiles) for YSD based on true GA.

**Supplementary Figure 4:**
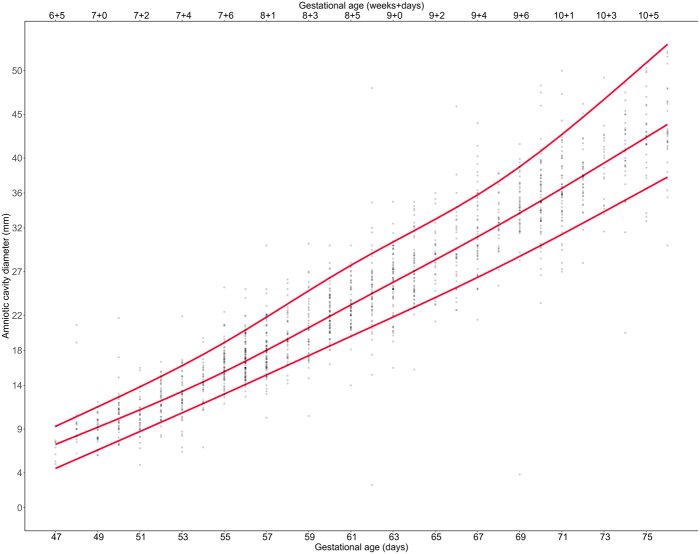
Mean amniotic cavity diameter measurements by gestational age with 10th, 50th, and 90th centiles for 716 assisted reproductive technology pregnancies (n = 1,320 measurements). *Grey dots* correspond to amniotic cavity diameter measurements of assisted reproductive technology pregnancies.

Supplemental Figure 4 illustrates scatter plots with the 10th, 50th, and 90th percentiles of ACD relative to GD. The chart covers the range from 6 + 5 to 10 + 6 gestational weeks and is based on 1,320 ACD measurements. Supplemental Table 4 presents reference values (10th, 50th, and 90th percentiles) for ACD based on true GA.

## Discussion

This study aimed to enhance the accuracy of pregnancy dating and assesses how dating based on the LMP affects growth curves in early pregnancy. The comparison of CRL growth chart based on LMP-dated spontaneous pregnancies with known GAs of ART pregnancies revealed a statistically significantly higher spread for the spontaneous pregnancies. Because the CRL-median of spontaneous pregnancies run above those of ART pregnancies, our data support the assumption that pregnancies are estimated to be more advanced for a given CRL dated by the LMP method than the real GA might be. Examining the 90th percentile, significant and clinically relevant distinctions emerge, with a GA estimated up to 4.08 days higher in spontaneous pregnancies than in ART pregnancies.

The increased variability of CRL reference values of spontaneous pregnancies compared with ART pregnancies is most likely to be attributed to inaccuracies stemming from the use of the LMP method. This technique is known to exhibit inaccuracies as indicated by prior studies (4, 6, 7).

The finding that the GA of LMP-dated pregnancies is estimated to be higher compared with the known GA of ART pregnancies confirmed the conclusions of earlier studies that have demonstrated an overestimation of the GA with the LMP method, potentially leading to the classification of an increased proportion of deliveries as post-term (6, 15, 16, 17).

The CRL growth chart based on ART pregnancies was compared with preexisting charts by Robinson/Fleming and Hadlock (8, 9). Within the mid-range, extending roughly from 7 + 0 to 9 + 0 gestational weeks, the two curves exhibited the closest alignment to the ART reference curve. The differences were more noticeable at very early GAs: the curve of Robinson/Fleming starts 10 GDs later than our novel growth chart based on known GA. There, it estimated the GA lower for a given CRL, possibly due to the fact that Robinson/Fleming only used transabdominal static ultrasound available at the time of their study (8). This limited technology might not have allowed for reliable detection of such small CRL sizes. This was in addition supported by the fact that Hadlock’s growth chart based on transvaginal measurements starts 4 days earlier than Robinson/Fleming’s chart.

The Hadlock curve overestimated the GA in the early first trimester compared with the ART pregnancies in our study. This pattern persisted consistently along most of the curve and became more pronounced as it approached the end of the observation at 10 + 6 gestational weeks. From 7 + 3 gestational weeks onward, the Robinson/Fleming curve closely resembled that of Hadlock. From this juncture, their estimations of GA in addition tended to be higher compared with what our curve indicated.

Because these preexisting curves were calculated based on the LMP method, we hypothesize that this influences their growth charts. As outlined above, the available literature indicates that the LMP method tends to overestimate GA. In agreement, the Robinson/Fleming and Hadlock growth curves tend to overestimate GA when compared with our growth chart, which relies on accurate timing of fertilization and embryonic implantation. Other factors may have contributed to the observed discrepancies, such as differences in ultrasound technology, disparities within the populations, and examiner-dependent factors. A systematic review of charts conducted by Napolitano et al. (18) revealed that discrepancies in GA estimation have been documented in both ART and spontaneous pregnancies, encompassing both under- and overestimations. The investigators speculated that these disparities were primarily due to variations in the methodologies employed in each of the studies.

Contrary to the curve of Robinson/Fleming and Hadlock and other more recent studies, we provide additional data in early pregnancy starting our CRL growth chart with 4 + 6 gestational weeks (7, 19, 20). As a result, with the newly calculated growth curves, GA is estimated approximately 1 week earlier than with the growth curves provided by Robinson/Fleming and Hadlock (8, 9).

Furthermore, this study incorporates additional biometric ultrasound variables such as GSD, YSD, and ACD. These variables have been shown to help detect early pregnancy complications and have been studied as prognostic factors for first-trimester outcomes (21, 22, 23, 24, 25). Until now, some studies produced CRL growth curves using known GA. However, in many instances, the dataset sample sizes were relatively modest, and these studies tended to focus solely on CRL measurements, without considering other variables like GSD, YSD, and ACD values (19, 20, 26, 27, 28). The most extensive study of an ART cohort regarding this matter has been conducted in a Chinese population (29). Therefore, our novel growth chart has the potential to become one of the most comprehensive charts for early pregnancy within a diverse population of ART pregnancies with regard to ethnic background, socioeconomic status, and infertility diagnosis. Furthermore, the GSD growth chart may be regarded as one of the most comprehensive GSD curves within an ART cohort, considering it encompasses 3,190 GSD measurements.

### Clinical implications

Dating by LMP may overestimate GA in early pregnancy. This may have implications in clinical settings, potentially affecting birth-management, such as the decision to induce labor, or playing a critical role in cases of premature birth approaching the viability threshold. We postulate that a growth chart originating from ART pregnancies may provide a more accurate means of estimating GA in comparison with growth charts based on spontaneous pregnancies dated using the LMP method. Because various biometric parameters appear crucial in predicting adverse pregnancy outcomes, our novel growth curves—covering not only CRL but also GSD, YSD, and ACD—may become useful for early pregnancy monitoring and research.

### Research implications

Prospective studies with predetermined analysis strategy and pre-established sample size calculations may aid in confirming these findings. Although our dataset of measurements must be considered extensive when compared with previous studies, our growth charts should undergo validation in even larger populations before widespread implementation in routine clinical practice.

### Strengths and limitations

The strengths of this study include the large dataset, close monitoring of the development of early pregnancies, and the application of clear and specific exclusion criteria to our study population. The use of data from ART pregnancies enabled us to use precisely known GAs to construct our growth curve. Other studies have also used the accuracy and reliability of dating based on the known date of conception in ART pregnancies to compare other methods of determining GA (30). We ensured the most accurate estimation of GA for the spontaneous pregnancies by limiting inclusion to women with regular menstrual cycles.

A limitation of our study is its single-center retrospective design, which focuses on a population of women undergoing infertility evaluation and treatment. The specific characteristics of this population, such as maternal age and underlying causes of infertility, may introduce potential selection bias. This could result in heightened perinatal risks and increased pregnancy complications that might impact fetal growth later in pregnancy but are unlikely to cause differences in growth during early pregnancy. Complications affecting early pregnancy growth, such as early or late abortions, chromosomal abnormalities, or fetal anomalies, were excluded from our study population. Our study’s descriptive statistics indicate no significant differences in preterm birth, fetal length, fetal head circumference, or birth weight between the ART and spontaneous pregnancy groups (31, 32). Besides, this potential bias enabled precise pregnancy dating in a large sample size at such an early GA. There are few studies on CRL growth in early pregnancy comparing ART and spontaneous pregnancies, but the existing research indicates similar growth patterns in both groups (33, 34, 35). One could in addition consider another limitation of our study that there are only data available up to 10 + 6 gestational weeks of pregnancy, thus limiting our reference curve to a certain time frame. However, substantial data already exist for measurements between 11 and 14 gestational weeks, so the chart presented here fills a gap in the existing literature.

## Conclusions

We present one of the most comprehensive charts for early pregnancy within a population of ART pregnancies comprising 2,803 CRL measurements with exact pregnancy dating. It reveals distinctions from the established growth curves commonly applied in daily practice. Additionally, we provide growth curves for parameters like GSD, YSD, and ACD. These novel and precise growth charts are expected to be highly relevant for management decisions during ongoing pregnancies.

## Declaration of Interests

J.H. has nothing to disclose. A.Q. has nothing to disclose. M.F. has nothing to disclose. A.S. has nothing to disclose. F.P. has nothing to disclose. C.D.G. has nothing to disclose. U.G.-K. has nothing to disclose. B.M. has nothing to disclose. G.M.-B. has nothing to disclose.
