# Supplementary material for: Novel sonographic reference charts for early pregnancy based on known gestational age
Source: F S Rep. 2024 Dec 4;6(1):52–9. doi: 10.1016/j.xfre.2024.11.008 (PMC11973808; doi:10.1016/j.xfre.2024.11.008)
Supplement: Supplementary data [file mmc1.docx]

**Supplementary Material**

**Supplemental table 1**: Median, 10th and 90th percentiles of crown-rump length for each gestational day ranging from 4 + 6 to 10 + 6 gestational weeks with the number of measurement points constituting the curve at each gestational day based on assisted reproductive technology pregnancies.

Abbreviations: GD=gestational age, NpGD=number of measurement points constituting the curve at each gestational day

| GD | 10th Percentile | Median | 90th Percentile | NpGD |
| --- | --- | --- | --- | --- |
| 34 | 1.01 | 1.30 | 1.77 | 8 |
| 35 | 1.10 | 1.48 | 2.11 | 30 |
| 36 | 1.19 | 1.67 | 2.46 | 42 |
| 37 | 1.29 | 1.86 | 2.80 | 37 |
| 38 | 1.40 | 2.06 | 3.16 | 59 |
| 39 | 1.54 | 2.31 | 3.56 | 91 |
| 40 | 1.74 | 2.62 | 4.00 | 61 |
| 41 | 2.00 | 3.00 | 4.51 | 75 |
| 42 | 2.35 | 3.48 | 5.10 | 98 |
| 43 | 2.80 | 4.08 | 5.80 | 125 |
| 44 | 3.36 | 4.81 | 6.60 | 85 |
| 45 | 4.02 | 5.65 | 7.50 | 85 |
| 46 | 4.77 | 6.59 | 8.47 | 99 |
| 47 | 5.60 | 7.59 | 9.50 | 89 |
| 48 | 6.48 | 8.63 | 10.56 | 64 |
| 49 | 7.41 | 9.70 | 11.63 | 69 |
| 50 | 8.37 | 10.77 | 12.69 | 86 |
| 51 | 9.34 | 11.81 | 13.72 | 45 |
| 52 | 10.31 | 12.81 | 14.70 | 55 |
| 53 | 11.28 | 13.77 | 15.65 | 61 |
| 54 | 12.25 | 14.71 | 16.57 | 48 |
| 55 | 13.24 | 15.64 | 17.48 | 73 |
| 56 | 14.23 | 16.56 | 18.39 | 99 |
| 57 | 15.25 | 17.49 | 19.32 | 85 |
| 58 | 16.27 | 18.44 | 20.29 | 49 |
| 59 | 17.32 | 19.43 | 21.29 | 47 |
| 60 | 18.40 | 20.45 | 22.36 | 80 |
| 61 | 19.50 | 21.53 | 23.50 | 68 |
| 62 | 20.63 | 22.68 | 24.73 | 62 |
| 63 | 21.80 | 23.90 | 26.05 | 74 |
| 64 | 23.00 | 25.19 | 27.46 | 67 |
| 65 | 24.23 | 26.54 | 28.96 | 36 |
| 66 | 25.48 | 27.96 | 30.53 | 40 |
| 67 | 26.76 | 29.42 | 32.17 | 61 |
| 68 | 28.05 | 30.93 | 33.86 | 44 |
| 69 | 29.36 | 32.47 | 35.61 | 71 |
| 70 | 30.69 | 34.05 | 37.40 | 92 |
| 71 | 32.03 | 35.66 | 39.22 | 74 |
| 72 | 33.38 | 37.28 | 41.07 | 50 |
| 73 | 34.73 | 38.91 | 42.93 | 36 |
| 74 | 36.09 | 40.56 | 44.80 | 51 |
| 75 | 37.44 | 42.20 | 46.67 | 68 |
| 76 | 38.80 | 43.84 | 48.55 | 64 |

**Supplemental table 2**: Median, 10th and 90th percentiles of gestational sac diameter for each gestational day ranging from 4 + 6 to 10 + 6 gestational weeks with the number of measurement points constituting the curve at each gestational day based on assisted reproductive technology pregnancies.

Abbreviations: GD=gestational age, NpGD=number of measurement points constituting the curve at each gestational day

| GD | 10th Percentile | Median | 90th Percentile | NpGD |
| --- | --- | --- | --- | --- |
| 31 | 1.19 | 2.00 | 4.11 | 6 |
| 32 | 1.93 | 3.00 | 5.30 | 18 |
| 33 | 2.66 | 4.00 | 6.49 | 11 |
| 34 | 3.40 | 5.00 | 7.68 | 23 |
| 35 | 4.14 | 6.01 | 8.87 | 88 |
| 36 | 4.87 | 7.01 | 10.06 | 137 |
| 37 | 5.61 | 8.01 | 11.25 | 106 |
| 38 | 6.35 | 9.01 | 12.44 | 118 |
| 39 | 7.10 | 10.03 | 13.65 | 134 |
| 40 | 7.86 | 11.05 | 14.86 | 73 |
| 41 | 8.65 | 12.09 | 16.09 | 79 |
| 42 | 9.46 | 13.14 | 17.33 | 99 |
| 43 | 10.29 | 14.21 | 18.60 | 126 |
| 44 | 11.15 | 15.30 | 19.89 | 85 |
| 45 | 12.03 | 16.40 | 21.19 | 85 |
| 46 | 12.93 | 17.51 | 22.50 | 99 |
| 47 | 13.85 | 18.63 | 23.83 | 89 |
| 48 | 14.78 | 19.75 | 25.16 | 65 |
| 49 | 15.71 | 20.87 | 26.49 | 69 |
| 50 | 16.65 | 21.99 | 27.83 | 86 |
| 51 | 17.60 | 23.10 | 29.16 | 45 |
| 52 | 18.55 | 24.20 | 30.48 | 54 |
| 53 | 19.49 | 25.29 | 31.80 | 61 |
| 54 | 20.43 | 26.37 | 33.11 | 48 |
| 55 | 21.38 | 27.44 | 34.41 | 73 |
| 56 | 22.32 | 28.50 | 35.69 | 99 |
| 57 | 23.27 | 29.55 | 36.96 | 85 |
| 58 | 24.22 | 30.60 | 38.22 | 49 |
| 59 | 25.16 | 31.63 | 39.45 | 47 |
| 60 | 26.11 | 32.66 | 40.67 | 79 |
| 61 | 27.06 | 33.68 | 41.87 | 67 |
| 62 | 28.02 | 34.69 | 43.05 | 62 |
| 63 | 28.97 | 35.69 | 44.20 | 73 |
| 64 | 29.93 | 36.69 | 45.33 | 67 |
| 65 | 30.89 | 37.68 | 46.44 | 36 |
| 66 | 31.85 | 38.67 | 47.54 | 41 |
| 67 | 32.81 | 39.65 | 48.61 | 60 |
| 68 | 33.77 | 40.63 | 49.68 | 44 |
| 69 | 34.73 | 41.60 | 50.73 | 70 |
| 70 | 35.70 | 42.57 | 51.77 | 92 |
| 71 | 36.67 | 43.54 | 52.80 | 74 |
| 72 | 37.63 | 44.51 | 53.83 | 50 |
| 73 | 38.60 | 45.47 | 54.86 | 36 |
| 74 | 39.57 | 46.44 | 55.88 | 50 |
| 75 | 40.53 | 47.40 | 56.90 | 68 |
| 76 | 41.50 | 48.36 | 57.92 | 64 |

**Supplemental table 3**: Median, 10th and 90th percentiles of yolk sac diameter for each gestational day with the number of measurement points constituting the curve at each gestational day (NpGD) based on assisted reproductive technology pregnancies.

Abbreviations: GD=gestational age, NpGD=number of measurement points constituting the curve at each gestational day

| GD | 10th Percentile | Median | 90th Percentile | NpGD |
| --- | --- | --- | --- | --- |
| 32 | 0.59 | 1.11 | 1.99 | 6 |
| 33 | 0.85 | 1.41 | 2.29 | 8 |
| 34 | 1.10 | 1.70 | 2.60 | 18 |
| 35 | 1.35 | 2.00 | 2.90 | 75 |
| 36 | 1.60 | 2.30 | 3.20 | 123 |
| 37 | 1.85 | 2.59 | 3.50 | 86 |
| 38 | 2.10 | 2.88 | 3.80 | 108 |
| 39 | 2.34 | 3.16 | 4.08 | 131 |
| 40 | 2.57 | 3.43 | 4.35 | 70 |
| 41 | 2.78 | 3.67 | 4.60 | 79 |
| 42 | 2.97 | 3.88 | 4.82 | 98 |
| 43 | 3.15 | 4.06 | 5.00 | 123 |
| 44 | 3.30 | 4.21 | 5.16 | 83 |
| 45 | 3.43 | 4.34 | 5.29 | 84 |
| 46 | 3.55 | 4.44 | 5.39 | 98 |
| 47 | 3.66 | 4.53 | 5.49 | 89 |
| 48 | 3.75 | 4.60 | 5.56 | 65 |
| 49 | 3.84 | 4.66 | 5.63 | 68 |
| 50 | 3.91 | 4.72 | 5.70 | 84 |
| 51 | 3.98 | 4.78 | 5.77 | 45 |
| 52 | 4.05 | 4.84 | 5.83 | 54 |
| 53 | 4.12 | 4.90 | 5.91 | 59 |
| 54 | 4.18 | 4.96 | 5.98 | 47 |
| 55 | 4.24 | 5.03 | 6.06 | 73 |
| 56 | 4.30 | 5.09 | 6.14 | 97 |
| 57 | 4.35 | 5.16 | 6.22 | 84 |
| 58 | 4.41 | 5.22 | 6.30 | 49 |
| 59 | 4.45 | 5.29 | 6.38 | 46 |
| 60 | 4.50 | 5.35 | 6.47 | 79 |
| 61 | 4.54 | 5.42 | 6.55 | 67 |
| 62 | 4.58 | 5.48 | 6.63 | 62 |
| 63 | 4.62 | 5.54 | 6.71 | 73 |
| 64 | 4.65 | 5.59 | 6.79 | 63 |
| 65 | 4.68 | 5.65 | 6.87 | 34 |
| 66 | 4.71 | 5.70 | 6.95 | 40 |
| 67 | 4.73 | 5.75 | 7.02 | 56 |
| 68 | 4.76 | 5.80 | 7.10 | 42 |
| 69 | 4.78 | 5.85 | 7.18 | 65 |
| 70 | 4.80 | 5.90 | 7.25 | 79 |
| 71 | 4.82 | 5.95 | 7.33 | 63 |
| 72 | 4.84 | 5.99 | 7.40 | 45 |
| 73 | 4.86 | 6.04 | 7.47 | 30 |
| 74 | 4.87 | 6.09 | 7.55 | 45 |
| 75 | 4.89 | 6.13 | 7.62 | 54 |
| 76 | 4.91 | 6.18 | 7.70 | 54 |

**Supplemental table 4**: Median, 10th and 90th percentiles of amniotic cavity diameter for each gestational day with the number of measurement points constituting the curve at each gestational day (NpGD) based on assisted reproductive technology pregnancies.

Abbreviations: GD=gestational age, NpGD=number of measurement points constituting the curve at each gestational day

| GD | 10th Percentile | Median | 90th Percentile | NpGD |
| --- | --- | --- | --- | --- |
| 47 | 4.49 | 7.24 | 9.30 | 9 |
| 48 | 5.55 | 8.22 | 10.42 | 15 |
| 49 | 6.60 | 9.20 | 11.54 | 29 |
| 50 | 7.66 | 10.19 | 12.67 | 38 |
| 51 | 8.71 | 11.20 | 13.82 | 31 |
| 52 | 9.78 | 12.23 | 15.01 | 40 |
| 53 | 10.84 | 13.29 | 16.24 | 46 |
| 54 | 11.92 | 14.39 | 17.53 | 33 |
| 55 | 13.00 | 15.54 | 18.89 | 63 |
| 56 | 14.09 | 16.75 | 20.32 | 92 |
| 57 | 15.19 | 18.00 | 21.80 | 74 |
| 58 | 16.29 | 19.28 | 23.31 | 41 |
| 59 | 17.39 | 20.59 | 24.82 | 41 |
| 60 | 18.50 | 21.90 | 26.30 | 66 |
| 61 | 19.60 | 23.20 | 27.73 | 55 |
| 62 | 20.71 | 24.50 | 29.09 | 56 |
| 63 | 21.81 | 25.79 | 30.40 | 64 |
| 64 | 22.92 | 27.07 | 31.69 | 56 |
| 65 | 24.04 | 28.36 | 33.00 | 30 |
| 66 | 25.18 | 29.66 | 34.35 | 34 |
| 67 | 26.34 | 30.98 | 35.78 | 48 |
| 68 | 27.53 | 32.32 | 37.32 | 30 |
| 69 | 28.75 | 33.70 | 39.00 | 47 |
| 70 | 30.00 | 35.10 | 40.80 | 67 |
| 71 | 31.27 | 36.53 | 42.70 | 53 |
| 72 | 32.56 | 37.97 | 44.67 | 37 |
| 73 | 33.87 | 39.43 | 46.71 | 22 |
| 74 | 35.18 | 40.90 | 48.80 | 32 |
| 75 | 36.50 | 42.37 | 50.90 | 35 |
| 76 | 37.82 | 43.85 | 53.00 | 36 |
